# Lipid Stores and Lipid Metabolism Associated Gene Expression in Porcine and Bovine Parthenogenetic Embryos Revealed by Fluorescent Staining and RNA-seq

**DOI:** 10.3390/ijms21186488

**Published:** 2020-09-05

**Authors:** Arkadiusz Kajdasz, Ewelina Warzych, Natalia Derebecka, Zofia E. Madeja, Dorota Lechniak, Joanna Wesoly, Piotr Pawlak

**Affiliations:** 1 Laboratory of Human Molecular Genetics, Institute of Molecular Biology and Biotechnology, Faculty of Biology, Adam Mickiewicz University, 61-614 Poznan, Poland; akajdasz@amu.edu.pl; 2 Department of Genetics and Animal Breeding, Faculty of Veterinary Medicine and Animal Sciences, Poznan University of Life Sciences, Wolynska 33, 60-637 Poznan, Poland; ewelina.warzych@up.poznan.pl (E.W.); zofia.madeja@up.poznan.pl (Z.E.M.); dorota.cieslak@up.poznan.pl (D.L.); 3 Laboratory of High Throughput Technologies, Institute of Molecular Biology and Biotechnology, Faculty of Biology, Adam Mickiewicz University, Umultowska 89, 61-614 Poznan, Poland; nataliad@amu.edu.pl (N.D.); j.wesoly@amu.edu.pl (J.W.)

**Keywords:** blastocyst, lipid droplet, parthenogenesis, lipid metabolism, gene expression, RNA-seq, pig, cattle

## Abstract

Compared to other mammalian species, porcine oocytes and embryos are characterized by large amounts of lipids stored mainly in the form of droplets in the cytoplasm. The amount and the morphology of lipid droplets (LD) change throughout the preimplantation development, however, relatively little is known about expression of genes involved in lipid metabolism of early embryos. We compared porcine and bovine blastocyst stage embryos as well as dissected inner cell mass (ICM) and trophoblast (TE) cell populations with regard to lipid droplet storage and expression of genes functionally annotated to selected lipid gene ontology terms using RNA-seq. Comparing the number and the volume occupied by LD between bovine and porcine blastocysts, we have found significant differences both at the level of single embryo and a single blastomere. Aside from different lipid content, we found that embryos regulate the lipid metabolism differentially at the gene expression level. Out of 125 genes, we found 73 to be differentially expressed between entire porcine and bovine blastocyst, and 36 and 51 to be divergent between ICM and TE cell lines. We noticed significant involvement of cholesterol and ganglioside metabolism in preimplantation embryos, as well as a possible shift towards glucose, rather than pyruvate dependence in bovine embryos. A number of genes like *DGAT1*, *CD36* or *NR1H3* may serve as lipid associated markers indicating distinct regulatory mechanisms, while upregulated *PLIN2*, *APOA1*, *SOAT1* indicate significant function during blastocyst formation and cell differentiation in both models.

## 1. Introduction

The patterns according to which the genes are expressed provide biological insight into their function. Tissue and species specificity, imprinting, methylation, temporal and allele specificity are just a few regulatory mechanisms describing regulation of gene expression in developmental biology. For over two decades, microarrays have been used to investigate expression of thousands of genes in different species depending on the development of technology and reference genomes. Over time, microarrays have been replaced by next generation sequencing platforms that enable sequencing of genomes on an unprecedented scale. In embryology, the breakthrough came with the work of Tang et al., who, for the first time, described global gene expression in mouse embryos at a single cell level by RNA-seq [1]. Moreover, the expression profile of individual blastomeres of human preimplantation embryos, as well as embryonic stem cell lines, were published in 2013 [2]. Since then, the RNA-seq technology revolutionized the embryology of model and laboratory animals as well as of livestock science. Nowadays pigs and cattle are considered to be the best animal models for biomedical applications. This is acknowledged due to the similarities in physiology and pathology on one hand, and the kinetics of preimplantation embryo development, timing of genome activation and blastocyst formation on the other [3].

Compared to other livestock animals, porcine oocytes and embryos are characterized by high lipid content stored mainly in the form of droplets in the cytoplasm [4,5,6,7,8]. Lipid droplets (LD) are dynamic organelles with important roles in lipid metabolism. LD consist largely of neutral lipids, predominantly triglyceride (TG) and cholesterol ester (CE) which provide substrates for energy production, membrane components and signaling lipids [9,10]. LD are thought to be formed de novo in the endoplasmic reticulum. Mature LD consist of a neutral lipid core coated with a phospholipid monolayer and structural proteins such as perilipins, regulators of LD function [11,12]. During mammalian oogenesis, LD are stored in the ooplasm, however, for unknown reasons, the exact content of LD varies widely among species [6,13]. Moreover, LD morphology changes dynamically during early embryonic development and in response to culture conditions [14,15].

Histochemical analysis of porcine embryos showed changes in lipid composition during preimplantation development, along with the observed differences between in vivo and in vitro derived embryos [16]. The LD content remained constant up to the morula stage and it decreased substantially during the blastocyst formation. With regard to bovine embryos, lipid content was observed to be constant up to the 8/16-cell stage, and was followed by a significant increase at the morula stage and a decrease in blastocysts [17]. Therefore, in both species, embryos at the blastocyst stage probably intensify β-oxidation to support increased energy demand during blastocyst formation, blastocoel expansion and hatching. It is supported by data summarized by Sturmey et al., who showed that in mice, cows and pigs, oxygen consumption greatly increases at the blastocyst stage [18]. In comparison to other species, the highest values of oxygen consumption were observed in pig embryos at all stages of preimplantation development. However, still relatively little is known about genes encoding factors involved in lipid metabolism during the preimplantation development. Significant amounts of lipids and hypothetical scenarios of lipid utilization at the time of implantation indicate their important function for early development. Therefore, unravelling the metabolic pathways and the genes involved in the processes of lipogenesis/lipolysis is crucial to understand the biology of preimplantation embryo as well as to optimize embryo culture conditions in vitro.

## 2. Results

### 2.1. Embryo Culture

Altogether, 186 and 214 porcine and bovine oocytes respectively were in vitro matured and activated for parthenogenetic development. A total of 89% (165/186) and 91% (197/214) of activated porcine and bovine oocytes cleaved within 24 h of culture. Blastocyst yield accounted for 36% for porcine (7 days postactivation—7 dpa) and 21% for bovine embryos (8 dpa). Mean number of blastomeres accounted for 30 (+/− 11) in porcine and 63 (+/− 17) in bovine embryos.

### 2.2. Lipid Droplet Characteristics

**Figure 1:**
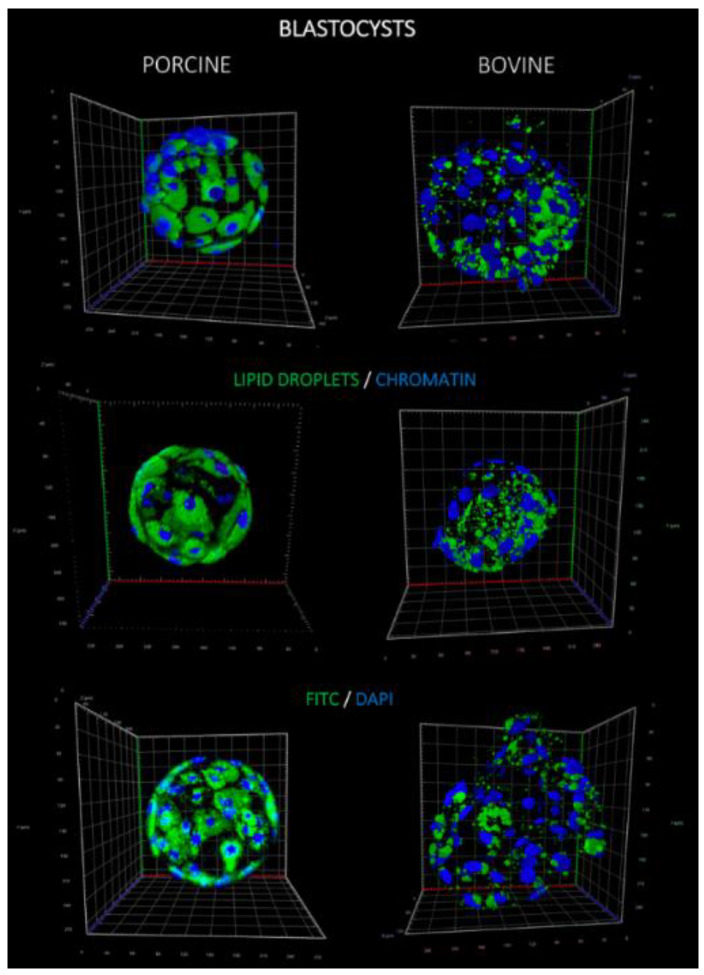
Confocal 3D projections of fluorescently stained porcine (left) and bovine (right) blastocyst stage embryos with BODIPY 493/503 (green) to visualize lipid droplets and DAPI (4′,6-diamidino-2-phenylindole; blue) for nucleus staining.

**Figure 2:**
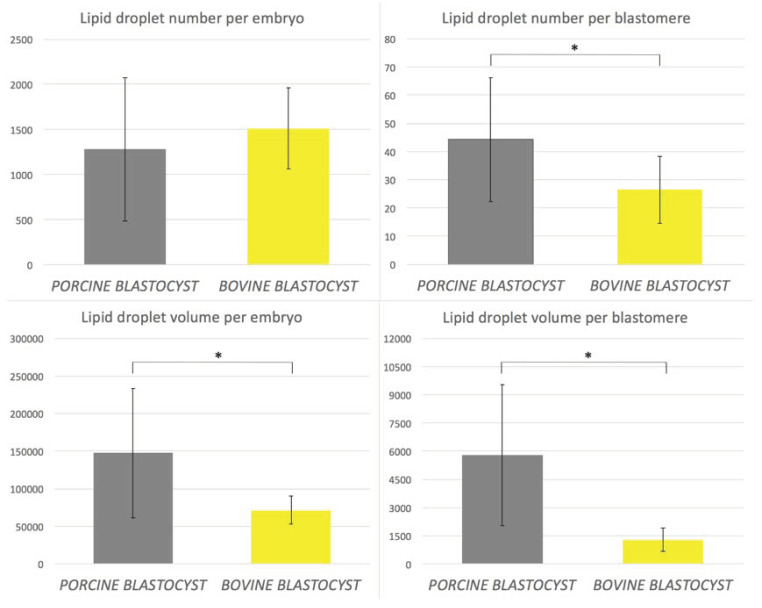
Lipid droplet number and volume calculated per single embryo and per blastomere in blastocyst stage embryos using Fiji software and 3D images captured with confocal microscope. Data are presented as mean +/− SD. Significant differences are marked with asterisks (* *p* < 0.05).

Altogether, 32 parthenogenetic blastocysts (18 porcine and 14 bovine) were analyzed for lipid storage (LD number and volume; Figure 1). Porcine embryos were abundantly packed with LD. Quantitative analysis revealed a positive correlation of lipid droplet number (per blastomere) with their volume only in bovine embryos (Figure 2 *p* < 0.01 *r* = 0.84). No correlation was found for porcine embryos and blastomeres which may result from heterogeneous population of LD differing in size. With regard to the entire embryo, a similar number of LD was present in bovine and porcine blastocysts, however, their volume was lower in bovine compared to pig embryos, due to morphological LD divergence between species (Figure 1 and Figure 2 *p* < 0.05). We observed significantly more LD per single blastomere in porcine embryos, a difference that arises from significantly higher number of cells in bovine embryos. According to our previous observations that LD number does not fully describe the lipid storage, we analyzed the volume occupied by LD in embryos and blastomeres. We noticed that LD exhibited significantly higher volume in porcine entire embryos and single blastomeres (Figure 2 *p* < 0.05).

### 2.3. RNA Sequencing of Porcine and Bovine Blastocyst Embryos

**Figure 3:**
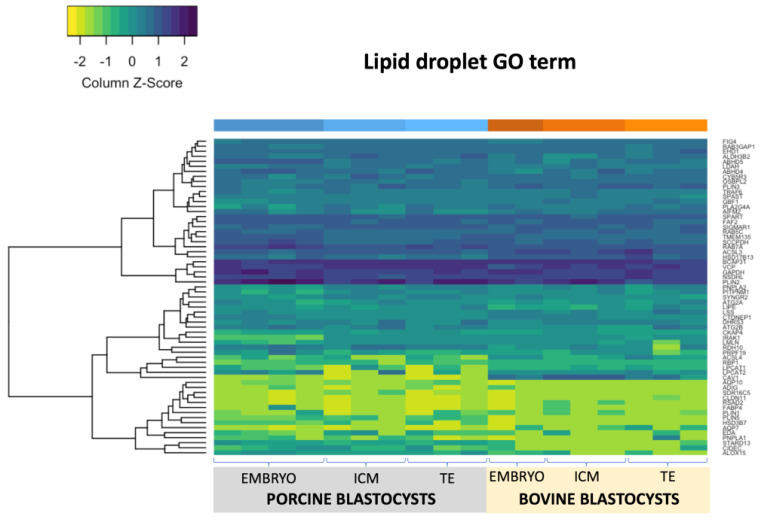
Heatmap of gene ontology (GO) term “lipid droplet” representing porcine and bovine parthenogenetic, blastocyst stage embryos, inner cell mass (ICM) and trophoblast (TE) cell lines.

**Figure 4:**
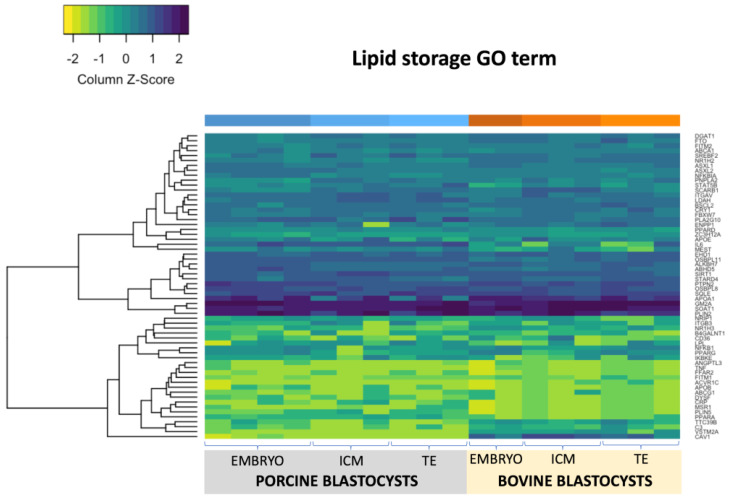
Heatmap of gene ontology (GO) term “lipid storage” representing porcine and bovine parthenogenetic, blastocyst stage embryos, ICM and TE cell lines.

Parthenogenetic embryos were collected at day 7 pa and day 8 pa of development for pig and bovine, respectively. Individual embryos as well as dissected inner cell mass (ICM) and trophoblast (TE) cell populations were sequenced. Altogether 18 samples have been analyzed (8 bovine and 10 porcine). The RNA-seq dataset has been deposited in GEO: accession GSE156149. The number of raw reads (pair-end) ranged from 22 to 65 million per sample for porcine embryos and from 34 to 53 million per sample for bovine. On average 11515 features (genes) were found after orthologues selection by NOIseq across the samples (Figure S1, Table S1). According to Gene ontology (GO) “lipid_droplet” term we found 44 out of 63 differentially expressed genes (DEG; *p* < 0.05; Figure 3) between whole porcine and bovine blastocyst; 19 between porcine and bovine ICMs and 23 between porcine and bovine TEs. Moreover, interspecies comparisons of ICM and TE populations showed 6 and 1 DEGs in bovine and porcine cell lines, respectively. A total of 17 genes were upregulated in porcine embryos, while bovine showed 27 genes significantly upregulated. With respect to blastocyst two cell lines we noticed 14 vs. 7 (ICM) and 10 vs. 12 (TE) significantly upregulated genes in porcine and bovine embryos, respectively. Gene ontology “lipid_storage” term with known 62 genes showed 34 DEGs between bovine and porcine blastocysts (*p* < 0.05; Figure 4). A total of 18 genes in whole porcine embryos and 14 genes in bovine were significantly upregulated, whereas ICM showed 19 and TE 23 DEGs. Among them, 13 vs. 6 (ICM) and 16 vs. 7 (TE) were significantly upregulated in porcine and bovine embryos, respectively.

**Figure 5:**
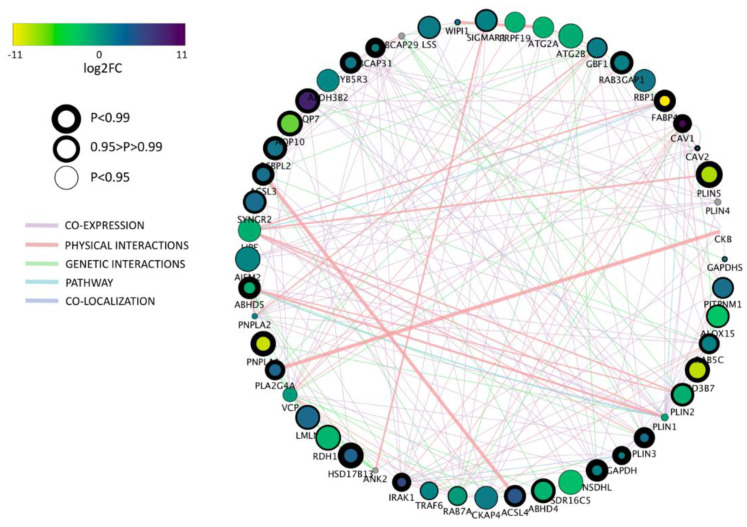
Network of differentially expressed genes (DEG) for GO term “lipid droplet” between porcine and bovine parthenogenetic, blastocyst stage embryos. Negative log2FC values indicate an upregulation of genes in porcine embryos. Node envelope thickness expressed as NOIseq probability value.

**Figure 6:**
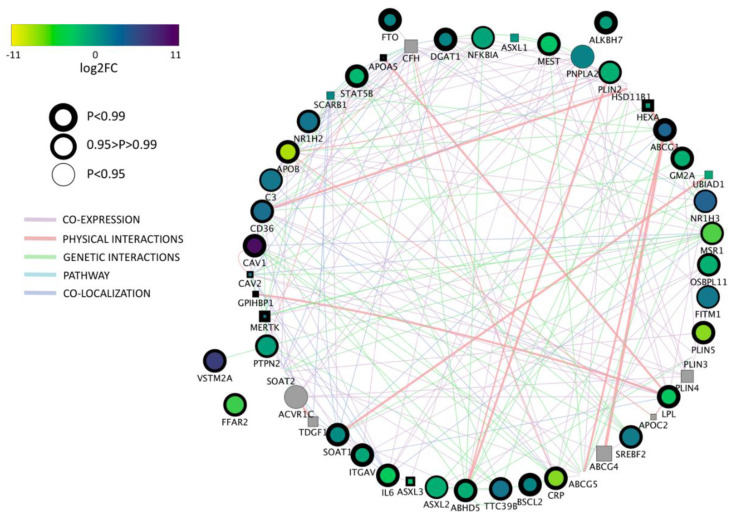
Network of differentially expressed genes (DEG) for GO term “lipid storage” between porcine and bovine parthenogenetic, blastocyst stage embryos. Negative log2FC values indicate an upregulation of genes in porcine embryos. Node envelope thickness expressed as NOIseq probability value.

In whole embryos, *GM2A*, *PLIN2*, *APOA1*, *SOAT1*, *SQLE*, *PTPN2*, *SIRT1*, *ALKBH7*, *OSBPL8, ABHD5*, *STEARD4* were the most abundantly expressed genes in both species (*APOA1*, *SQLE*, *SIRT1*, *OSBPL8* were not differentially expressed). *PLIN2*, *GM2A* and *SOAT1* expression was outreach among other DEGs (Figure 5 and Figure 6). In addition to these genes, we found *CD36*, *SCARB1* and *CAV1* abundantly expressed in the bovine ICM samples. TE cells abundantly expressed *IL6* gene, which was upregulated in porcine samples.

## 3. Discussion

Lipids play a fundamental role in development of preimplantation embryos by providing energy and other factors, such as hormone precursors. Staining of lipid droplets revealed that both the number of LD per single blastomere and the LD volume were significantly higher in porcine blastocysts, in comparison to bovine. This observation may be supported by the distribution of LD in oocytes of both species, since significantly higher lipid content in porcine oocytes was noticed when compared to, e.g., bovine, human or murine counterparts [6]. Yet, the functional and biological relevance of high lipid stores in porcine oocytes and embryos still remains to be solved. In our research, we have used a model of parthenogenetic embryo development, which is routinely used in the in vitro porcine embryo production, and also serves as a source for the derivation of embryonic stem cell lines.

Based on the gene ontology (GO) term selection on “lipid droplet” we found 69.5% (44 among 63) differentially expressed genes between bovine and porcine parthenogenetic blastocysts. Among them, *PLIN2* and *PLIN3* were found which play crucial roles in LD metabolism. *PLIN2* prevents lipid degradation and hence promotes lipid accumulation, whereas *PLIN3* can be recruited to the newly synthesized LD, suggesting its involvement in protection and trafficking of the nascent LD [11,19]. The results of our experiment showed higher expression of the *PLIN2* gene in porcine blastocysts and, on the contrary, an upregulation of *PLIN3* in bovine blastocysts. Since blastomeres of porcine blastocysts already exhibit high accumulation of lipids (as shown by fluorescent staining), recognizing high level of *PLIN2* as a marker of cellular lipid accumulation is justified. Bovine blastocysts, on the other hand, were characterized by lower lipid accumulation. Thus, it may be anticipated that a prior activation of mechanisms, which increase the accumulation of energy sources may be required. This process may be controlled by the product of the *PLIN3* gene associated with the newly formed LD. This observation may be supported by higher number of LD noted in bovine blastocysts calculated per whole embryo. The probable higher dynamics of LD formation in the bovine blastocysts may also be supported by higher expression of the *NSDHL* gene. Its product is involved in synthesis of cholesterol, which after esterification is one of the main components of the LD core [20]. Moreover, we have observed higher expression of the *ACSL3* gene in bovine blastocysts, which is involved in the generation of long-chain acyl-CoA and modulates cellular fatty acid uptake [17]. Paczkowski et al. suggested that upregulation of *ACSL3* may be an indicator of fatty acid oxidation (FAO) rise [21]. Therefore, the data described above indicates, that although bovine blastocysts contain less LD, their formation and FAO is more dynamic at the blastocyst stage when compared to porcine embryos.

One of the most intriguing finding concerns caveolin 1 (*CAV1*) expression in porcine and bovine blastocysts. Caveolin-deficient mouse adipocytes show disturbed LD formation, different protein and lipid composition, as well as a different size distribution of LD. It is suggested that caveolins seem to play an important role in LD maturation and biogenesis [22,23]. Knockout mice of *CAV1* gene showed reduced whole body FAO [24]. Additionally, other experiments indicated that the loss of *CAV1* (lack of expression) causes a switch from lipid toward glucose metabolism [25,26]. Since we have observed a very low level of caveolin 1 transcript in porcine blastocysts, it might suggest that these embryos may trigger the energy metabolism preferably towards glucose consumption. This assumption may be additionally corroborated by the upregulation of genes engaged in glucose metabolism observed in porcine blastocysts. We show significantly higher expression of glucose transporter 2 (*GLUT2*), as well as hexokinase 2 (*HEX2*) or *PDHA1* genes in porcine vs. bovine blastocysts. These genes are important for transport and conversion of glucose and their higher expression indicates the increased utilization of glucose as an energy source. Unlike pig embryos, bovine parthenogenetic blastocysts expressed a high level of *CAV1* transcript, confirming their constant dependency on fatty acids as an energy source. However, low mRNA level of genes controlling glucose metabolism (*HEX2*, *GLUT2*) may indicate limited consumption of glucose by embryos of this species. From our data, it may be concluded that bovine parthenogenetic blastocysts set their energy metabolism rather on fatty acids, whereas porcine parthenogenetic blastocysts switch into glucose consumption. Moreover, it was previously suggested that the energy state of the embryo does not have to be solely granted to either glucose or fatty acids, but it might function as a fine-tuned adaptive mechanism, reliable on multiple environmental and embryo originating factors.

Blastocyst formation is characterized by differentiation of blastomeres into pluripotent inner cell mass (ICM) that will give rise to the embryo proper and trophectoderm (TE) differentiating to the extraembryonic tissues. The two cell lines express different lineage specific markers, however, little is known about the differences related to the functional metabolic pathways. Here, we report that porcine ICM and TE cells do not show differences in gene expression with respect to “lipid storage”, and only one gene with very low expression level in “lipid droplet” GO terms. On the contrary, we found significant differences in gene expression between bovine ICM and TE with eight genes related to “lipid droplets” GO term and 23 for “lipid storage”. In porcine blastocysts, only the *PLIN1* gene was significantly different, showing higher expression in TE cells however, the expression was very low in both cell lines. *PLIN2* was one of the most abundantly expressed gene in ICM and TE of both species. Although the expression was comparable in ICMs, the TE cells upregulated *PLIN2* in porcine embryos, compared to bovine, which correspond to the overall LD number. Morphologically, porcine embryos have a lower number of blastomeres than bovine, and the ICM may be considered “non-obvious” due to strong accumulation of LD in embryo compartments. Therefore, more relevant differences may be found at later stages of development of porcine embryos, while in cattle, the ICM is already clearly established and easy to distinguish. Although the expression was low in bovine and porcine embryos, we noticed the presence of *PLIN5* transcripts and upregulation in ICM and TE cells in pig, which may indicate its role in LD biogenesis in preimplantation embryos.

Considering the results obtained from fluorescence staining, we also analyzed “lipid storage” gene ontology term, which shares with GO “lipid droplet” only seven of 125 genes. We found the highest expression of *GM2A* (GM2 ganglioside activator) gene, significantly upregulated in porcine embryos. GM2 Activator, also known as SAP3 (Shingolipid Activator Protein 3), coded by the *GM2A* gene, is one of the components of the B-hexosaminidaseA (*HEXA*) complex necessary for the enzymatic processing of gangliosides, which are sialic acid-containing acidic glycosphingolipids. Gangliosides found in animal plasma membranes exhibit cell-type specificity and are implicated in various cellular functions, differentiation, signal transduction and immunity [27,28]. The *HEXA* gene in our study was also significantly higher expressed in porcine blastocysts which corresponds to upregulation of a substrate specific cofactor *GM2A*. Most of publications describe GM1, GM3 and GD1a gangliosides as important factors involved in the EGFR (epidermal growth factor receptor) signaling pathway [28]. In porcine embryos, exogenous GD1a supported maturation and blastocyst formation in vitro [29]. Others report *GM1* and *GM3* expression in theca and cumulus rat cells, while Ju et al. correlated *GM3* expression with apoptosis in preimplantation mouse embryos [30]. To our knowledge, this is the first report on abundant expression of *GM2a* in porcine and bovine preimplantation embryos. Due to known antioxidative, signal transduction and cell differentiation functions of gangliosides, the upregulation of *GM2a* may indicate relevant function during the time of ICM and TE formation. According to BioGRID, a genetic interaction (GeneMania) exist between *HEXA* and *ABHD5* (also known as *CGI-58*), which is responsible for activation of *PNPLA2* lipase after dissociation from phosphorylated perilipin on LD surface. Although *ABHD5* was upregulated in porcine blastocyst, *PNPLA2* lipase displayed similar expression level. Interestingly, LD associated hydrolase (*LDAH*) expression was increased in porcine ICM and TE cell lines, compared to bovine. It has been recently shown that *LDAH* promote TAG accumulation and fusion of lipid droplets facilitating the metabolism towards lipid storage [31]. These results support the conclusion of Goo et al. that *LDAH* plays primarily lipogenic role together with the inhibition of *PNPLA2*. The second the most abundantly expressed gene was *SOAT1* (Sterol O-Acyltransferase 1). In mouse, microarray data showed that *SOAT1* was one of the genes which expression significantly increased during morula to blastocyst transition, what may be related to the differentiation of the first two embryonic cell lineages [32]. The Soats genes contribute to cholesterol metabolism mainly by esterification to enable efficient uptake and transportation. It is believed that *SOAT1* plays major role in maintenance of cholesterol homeostasis in cells and contribute to the accumulation of LD which is in line with our observations [33]. In our study, abundant *SOAT1* expression was probably reflected with high expression of *APOA1*, a component of high-density lipoprotein (HDL) that promotes cholesterol efflux. Taking these results and the already mentioned upregulation of the *NSDHL* gene in bovine blastocyst together, we suggest a significant role of cholesterol metabolism during preimplantation development.

In conclusion, we have described significant differences in lipid metabolism between bovine and porcine parthenogenetic blastocysts. Aside from differences in lipid stores reflected by the LD number and volume, the parthenotes of both species also regulate lipid metabolism differentially on the gene expression level. Some genes, like *CAV1 or GMA2*, may be considered species specific and stage specific markers of the embryo metabolism. Other, such as *FAB4*, *DGAT1*, *CD36* or *NR1H3*, although not highly expressed, show significant different between porcine and bovine embryos. This observation may therefore indicate lipid associated markers pointing to distinct regulatory mechanisms, which include cytoplasmic and nuclear factors. Genes characterized by the highest expression in both species, e.g., *GMA2*, *PLIN2*, *APOA1*, *SOAT*, *GAPDH* or *BCAP31*, may suggest their superior role in the regulation of metabolic pathways during blastocyst formation and cell differentiation.

## 4. Materials and Methods

All procedures were performed in accordance with the “Act on the protection of animals used for scientific purpose” of the Republic of Poland, which complies with the European Union Legislation for the protection of animals used for scientific purposes (Dz.U. 2015 poz. 266; 15 January 2015). According to these regulations ethics approval was not required, as the biological material (ovaries) was collected upon animal slaughter in abattoir (porcine—Sokolow S.A. Robakowo; bovine—Biernacki Sp. z o.o., Golina). Unless stated otherwise, all chemicals were purchased from Sigma.

### 4.1. Collection of Cumulus-Oocyte Complexes

The cumulus-oocyte complexes (COCs) were collected post-mortem from bovine and porcine ovarian follicles of 3–6 mm diameter. Ovaries were transported in thermoisolated flask 2–4 h after slaughter to the laboratory. Ovaries were collected from pubertal gilts and heifers. Immature COCs were aspirated from ovarian follicles with needle and syringe and scored morphologically in HEPES-Talp medium. Only COCs presenting evenly granulated cytoplasm and at least 3–4 layers of cumulus cells were used in experiment.

### 4.2. In Vitro Maturation

In vitro maturation of porcine COCs was performed in 500 μL of IVM medium (NCSU-23; North Carolina State University Medium-23) in four-well plates (Nunc, NY, USA). Each 500 μL IVM medium consisted of 70 COCs that were matured in HeraCell 150 incubator (Thermo Scientific, MA, USA) under conditions: 5% CO_2_ in atmosphere, 38.5 °C and maximum humidity. The first 24 h of porcine IVM included medium supplemented with hormones: 10 U PMSG (pregnant mare serum gonadotropin, Chorulon, MSD Animal Health, NL, USA) and 10 U hCG (Folligon, MSD Animal Health, NL, USA). The next step included the transfer of COCs to fresh, equilibrated medium without hormones for 20 h maturation. The same batch of follicular fluid (10% *v*/*v*) was used throughout the whole experiment. Bovine COCs were matured at 39 °C, 5% CO_2_ at maximum humidity in TCM199 medium supplemented with 0.006 g/mL fafBSA, 0.25 mM Na-pyruvate, 1× conc. penicillin-streptomycin, 0.02 U/mL LH, 0.002 mg/mL FHS and 0.001 mg/mL β-estradiol for 24 h. After IVM, COCs were denuded and all oocytes with first polar body, and no signs of degeneration were activated for parthenogenetic development.

### 4.3. Embryo Culture

Denuded oocytes were activated using 5 μM ionomycin in TALP-medium (5 min) and incubated in 2 mM 6-DMAP for 4 h in final embryo culture medium supplemented with BSA (for the prevention of the second polar body extrusion). NCSU23 and SOF were used as embryo culture media up to blastocyst stage (7th day post activation for porcine embryos and 9th day for bovine). At day 5 of porcine embryo development, a half of the medium was changed with fresh medium, equilibrated and supplemented with 20% of FBS. At the 3rd day of bovine embryo development half of the medium was changed with fresh and equilibrated SOF. The blastocyst rate was calculated in relation to activated oocytes.

### 4.4. Lipid Droplets Staining

Porcine and bovine blastocysts were fixed in 4% PFA for 30 min at 37 °C in four-well plates (Nunc). PFA was removed by washing the embryos twice in PBS with 0.2% PVP and stored at 4 °C for no longer than two weeks. Embryo permeabilization was performed with 0.2% Triton X-100 solution for 30 min at room temp. (RT) and washed 2× afterwards in 0.2% PVP/PBS. Lipid droplets were stained with fluorescent dye—20 μg/mL BODIPY 493/503 (Thermo Scientific, Carlsbad, CA, USA) at room temperature for one hour. The chromatin of blastomeres were visualized by staining with 0.5 μg/mL DAPI (4′,6-diamidino-2-phenylindole; Vector Laboratories, Burlingame, CA, USA). Embryos were mounted on glass slide with single concave (Comex, PL), coverslipped and analyzed using confocal microscope Zeiss LSM 880 using 488 nm filter with band pass 500–550 nm for BODIPY 493/503 (Laser Argon2) and 420–480 nm for DAPI (Laser Diode 405). LD were assessed through several optical sections (Z-stack) captured every 5 μm to exclude double positioning of the same structures on two stacks. Objective (LD LCl Plan Apochromat 40×/1.2 Imm Korr DIC 27; Zeiss, Germany), pinhole, filters, offset settings were kept constant throughout the experiments. LD counting using ImageJ software (NIH, Bethesda, MD, USA) was previously described.

### 4.5. Sample Collection for RNA-seq

Single porcine and bovine blastocyst stage embryos were selected from culture media and washed 3× in prewarmed PBS (ThermoFisher Scientific). SMART-Seq v4 Ultra Low Input RNA Kit for Sequencing (Clontech Takara) was used to generate high-quality cDNA from samples. The kit was designed to work with ultra-low amounts of total RNA (also at single cell level) and full-length transcripts. Samples were prepared according to manufacturer protocol. Firstly, 10× reaction buffer was prepared by combining 10× lysis solution and RNAse inhibitor. Embryos were transferred to 3 μL of ultrapure water (Ambion, Austin, TX, USA) in 96 LoBind, semi-skirted Eppendorf twin.tec^®^ PCR clean plate in the volume of 6.5 μL PBS. Immediately 1 μL of freshly made reaction buffer was added to embryos, vortexed gently and incubated at room temp. for 5 min. Next, 3′ SMART-Seq CDS Primer II (2 μL) was added to each sample, covered with foil and incubated for 3 min. in 72 °C using Biometra thermocycler (TProfessional). Next a reaction mixture for first strand cDNA synthesis was added to each sample in the volume of 7.5 μL and consisting of: 2 μL SMARTScribe Reverse Transcriptase, 1 μL of SMART-Seq v4 Oligonucleotide (48 μM), 4 μL of 5× Ultra Low First-Strand Buffer, 0.5 μL of RNase Inhibitor (40 U/μL). The reaction steps included 42 °C incubation for 90 min. and 70 °C incubation for 10 min. followed by transfer of plate on ice. Directly after cDNA synthesis it was amplified by LD PCR. A mixture of 25 μL 2× SeqAmp PCR Buffer, 1 μL PCR Primer II A (12 μM), 1 μL SeqAmp DNA Polymerase and 3 μL Nuclease-Free water was added to each cDNA sample. The shaken and spinned plate was placed back into the Biometra thermocycler with the following protocol setup: initial denaturation: 95 °C/1 min., amplification (17 cycles): 98 °C/10 s, 65 °C/30 s, 68 °C/3 min., final extension in 72 °C for 10 min., followed by 4 °C storage until purification. cDNA was further cleaned and eluted prior quality check and library preparation. According to Clontech protocol a 50 μL Agencourt AMPure XP beads and 1 μL of lysis buffer were used at first to bind cDNA in final reaction volume for 10 min at room temperature. The 96-plate was placed on a magnetic stand (Ambion, Life Technologies) for 10 min., and the remaining solution was discarded. A freshly made 80% pure ethanol solution was applied to each sample well twice to wash the cDNA. Washing steps lasted for 2 min, with the plate remaining on the magnetic stand. Finally, samples were air dried for 2–3 min. and re-suspended in 17 μL of Elution Buffer. Samples were pipetted few times in order to ensure complete elution and dissociation from magnetic beads. The plate was placed on a magnetics stand for 5 min., and the eluent was transferred to a fresh Low Bind 0.5 ml Eppendorf tube.

### 4.6. Library Preparation

Double stranded cDNA was prepared from total RNA isolated from each sample with the use of SMARTer Ultra Low RNA Kit for Illumina Sequencing (Clontech Takara), according to user manual with 17 cycles of amplification. Final libraries were prepared from cDNA with the use of the Nextera XT DNA library preparation kit and Nextera XT Index Kit v2 (Illumina, San Diego, CA, USA). Quality and quantity of cDNA for each sample were assessed with Agilent Bioanalyzer and Qubit fluorometer. Library preparation was performed according to manufacturer instruction for single-cell workflow with 500 pg cDNA input (according to Qubit measurements) and 12 cycles of amplification. The size of final library constructs was assessed by means of on-chip electrophoresis with the use of Agilent Bioanalyzer and Agilent High Sensitivity DNA Kit. The concentration of libraries was measured by the use of a Qubit 4 fluorometer and QUBIT dsDNA HS assay (ThermoFisher Scientific). All final libraries were diluted with 10 mM Tris-Cl to 2 nM, and then pooled by volume. Libraries were validated by shallow sequencing on Illumina MiSeq^®^ System with the use of MiSeq^®^ Reagent Kits v2 (500 cycles). A validation run was performed in 2 × 250 bp PE mode with 10 pM libraries and 5%PhiX spiked-in. The library pool was sequenced with 2 × 150 PE mode (NovaSeq™6000, Illumina, San Diego, CA, USA).

### 4.7. mRNA Gene Expression

qPCR was done using the standard curve method. For this purpose, the desired sequences for analyzed genes were amplified by PCR and visualized on 1.5% agarose gel with the Gene Ruler^TM^ 100 bp DNA Ladder (Fermentas, Burlington, ON, Canada). The PCR product was extracted from the gel, isolated and purified using the Gel Extraction Kit (Fermentas, Burlington, ON, Canada). Based on the DNA concentration measured with a Nanodrop c2000 system (Thermo Scientific, Carlsbad, CA, USA), a serial 10-fold dilutions of a DNA with a known concentration (standards) were generated. Each standard was used as a separate template for a real-time PCR reaction to produce the appropriate standard curve with the LightCycler 480 II software (Roche, Basel, Switzerland). The reaction conditions and the efficiency of the reactions for all genes were analyzed separately. All reactions were performed using the Light Cycler 480 II system with a set of supplied reagents. The 10 μL reaction mixture consisted of 5 μL of the LightCycler Probe Master, 0.5 μM primers, 0.3 μM probes and 1 μL each of the cDNA. The reaction conditions were as follows: denaturation: 95 °C, 10 min; amplification: 40 cycles of 95 °C for 10 s, 60 °C for 10 s, 72 °C for 10 s; and final cooling at 40 °C. The temperature slope was set at 20 °C/s during amplification.

### 4.8. Bioinformatic Tools, Data Processing and Statistics

Raw reads of *Sus scrofa* and *Bos taurus* were aligned to Ensembl Sscrofa11.1 and ARS-UCD1.2 genomes, respectively, with STAR (version 2.7). Reads mapped to exons were counted with featureCounts (version 1.6). List of orthologous genes in pigs and cows were obtained from the Ensembl database. For further analysis, we included orthologue genes with one-to-one matching and excluded duplicates, unmatched, one-to-many matching genes (16,384 genes left). Orthologue genes with no expression were removed from the analysis (15,023 genes remaining). Row reads were normalized with SCnorm package (version 1.8). Differential genes expression was calculated with NOISeq package (version 2.30). Heatmaps were created with M3Drop (version 1.12) and/or gplots (version 3.0) with basic plot functions of R. All R steps were performed in R version 3.6. Lists containing genes belonging to “lipid droplet” (GO:0005811), and “lipid storage” (GO:0019915) gene ontology terms were downloaded from the AmiGO 2 database. The selected, differentially expressed genes between porcine and bovine blastocysts of both GO terms have been uploaded to DAVID Bioinformatics Resources 6.3 and analyzed using a functional annotation tool. The minimum number of genes for the corresponding term was set as 3. EASE Score refers to the one-tail Fisher exact probability value, and was set as 0.05. A comparison of LD characteristics between experimental groups was performed using IBM SPSS Statistics 23.0. All data (before computing) were subjected to testing for normal distribution using the Kolmogorov-Smirnov and Shapiro-Wilk tests. The differences in lipid droplet number and occupied volume in embryos were calculated using the Kruskal-Wallis and two-tailed Mann-Whitney U tests. Pearson correlation coefficient was calculated for lipid droplet number and volume association. All data with *p* < 0.05 were considered statistically significant.

## Supplementary Materials

The following are available online at https://www.mdpi.com/1422-0067/21/18/6488/s1.

## Abbreviations

LD	Lipid droplet
NCSU23	North Carolina State University 23 Medium
SOF	Synthetic Oviduct Fluid Medium
ICM	Inner Cell Mass
DMAP	(Dimethylamino)purine
TE	Trophectoderm
DEG	Differentially expressed gene
DAPI	4′,6-diamidino-2-phenylindole
FAO	Fatty acid oxidation
