# Supplementary material for: Lipid Stores and Lipid Metabolism Associated Gene Expression in Porcine and Bovine Parthenogenetic Embryos Revealed by Fluorescent Staining and RNA-seq
Source: Int J Mol Sci. 2020 Sep 5;21(18):6488. doi: 10.3390/ijms21186488 (PMC7555686; doi:10.3390/ijms21186488)
Supplement: Supplementary file 1 [file ijms-21-06488-s001.pdf]

Supplementary Materials:

Article

Lipid Stores and Lipid Metabolism Associated Gene Expression in Porcine and Bovine Parthenogenetic Embryos Revealed by fluorescence staining and RNA-seq

Arkadiusz Kajdasz [3], Ewelina Warzych [2], Natalia Derebecka [1], Zofia E Madeja [2], Dorota Lechniak [2], Joanna Wesoly [1] and Piotr Pawlak [2,*]

[1] Laboratory of High Throughput Technologies, Institute of Molecular Biology and Biotechnology, Faculty of Biology, Adam Mickiewicz University, Umultowska 89, 61-614 Poznan, Poland; nataliad@amu.edu.pl (N.D.); j.wesoly@amu.edu.pl (J.W.)

[2] Department of Genetics and Animal Breeding, Faculty of Veterinary Medicine and Animal Sciences, Poznan University of Life Sciences; Wolynska 33, 60-637 Poznan, Poland; ewelina.warzych@up.poznan.pl (E.W.); zofia.madeja@up.poznan.pl (Z.M.); dorota.cieslak@up.poznan.pl (D.L.); piotr.pawlak@up.poznan.pl (P.P.)

[3] Laboratory of Human Molecular Genetics, Institute of Molecular Biology and Biotechnology, Faculty of Biology, Adam Mickiewicz University, 61-614 Poznan, Poland; akajdasz@amu.edu.pl (A.K.);

* Correspondence: piotr.pawlak@up.poznan.pl; Tel.: +48-61846-6111

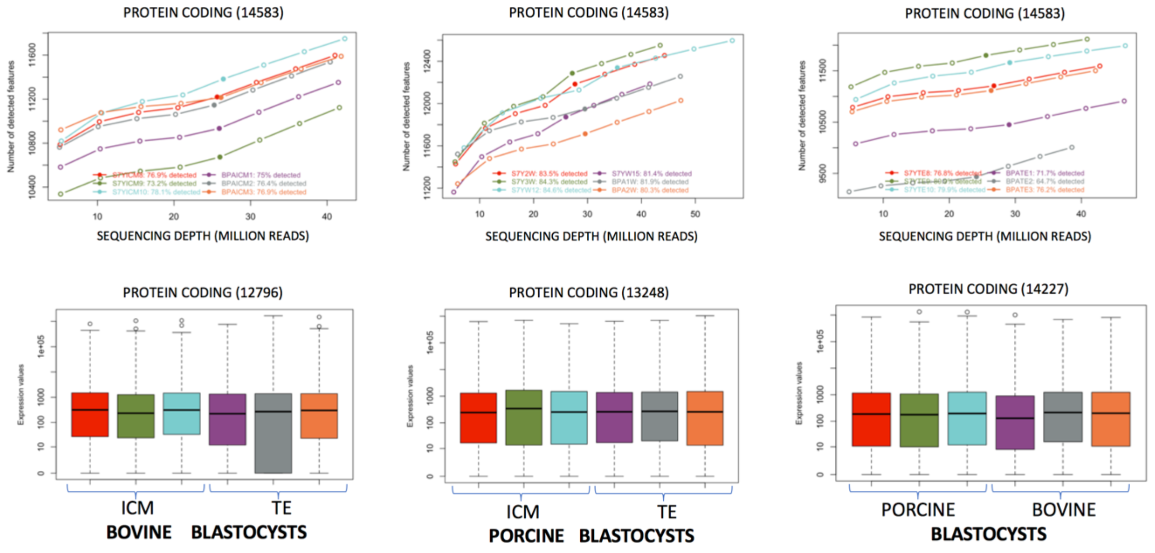

Supplementary Figure S1. Upper panel. Saturation plots for protein-coding genes identified in RNA-seq in all samples. Plots show number of detected features in sequencing depth context with lower and higher simulated RNA-seq depths. Lower panel. Distribution of protein coding gene counts per sample.

Supplementary Table S1. List of samples representing RNA-seq quality data, raw reads, alignment scores and normalization data after orthologue selection. S – sus scrofa (pig), B – bos taurus (bovine), ICM – inner cell mass, TE – trophectoderm, W – whole blastocyst

				Orthologues selection
Sample name	Raw reads	% uniquely mapped reads (*STAR*)	% of exonic counts (*featureCounts*)	Total features (*NOISeq*)
S2W	26 310 662	91.8	73.0	12 357
S3W	31 533 925	89.9	69.8	12 462
S4W	65 080 669	88.6	68.0	12 548
S5W	31 986 312	93.4	75.8	12 042
SICM1	22 913 077	93.4	73.0	11 371
SICM2	29 594 241	92.0	75.1	10 769
SICM3	38 800 003	94.8	78.0	11 539
STE1	24 104 932	93.3	73.0	11 357
STE2	29 126 629	94.7	78.5	11 960
STE3	44 296 009	94.8	79.9	11 835
B1W	36 653 697	83.5	73.9	12 129
B2W	38 202 533	71.4	74.7	11 879
BICM1	34 034 686	76.2	80.8	11 051
BICM2	51 748 198	83.7	80.3	11 311
BICM3	53 032 000	84.4	82.8	11 344
BTE1	36 277 963	72.4	75.0	10 548
BTE2	48 001 665	83.7	82.3	9 525
BTE3	43 608 460	75.6	80.8	11 253
